# Development of a gridded meteorological dataset over Java island, Indonesia 1985–2014

**DOI:** 10.1038/sdata.2017.72

**Published:** 2017-05-23

**Authors:** Yanto, Ben Livneh, Balaji Rajagopalan

**Affiliations:** 1 Civil Engineering Department, Jenderal Soedirman University, Purwokerto 53371, Indonesia; 2 Department of Civil Environmental and Architectural Engineering, University of Colorado, Boulder, Colorado 80309, USA; 3 Cooperative Institute for Research in Environmental Sciences, University of Colorado, Boulder, Colorado 80309, USA

**Keywords:** Environmental sciences, Water resources, Climate change, Hydrology

## Abstract

We describe a gridded daily meteorology dataset consisting of precipitation, minimum and maximum temperature over Java Island, Indonesia at 0.125°×0.125° (~14 km) resolution spanning 30 years from 1985–2014. Importantly, this data set represents a marked improvement from existing gridded data sets over Java with higher spatial resolution, derived exclusively from ground-based observations unlike existing satellite or reanalysis-based products. Gap-infilling and gridding were performed via the Inverse Distance Weighting (IDW) interpolation method (radius, r, of 25 km and power of influence, α, of 3 as optimal parameters) restricted to only those stations including at least 3,650 days (~10 years) of valid data. We employed MSWEP and CHIRPS rainfall products in the cross-validation. It shows that the gridded rainfall presented here produces the most reasonable performance. Visual inspection reveals an increasing performance of gridded precipitation from grid, watershed to island scale. The data set, stored in a network common data form (NetCDF), is intended to support watershed-scale and island-scale studies of short-term and long-term climate, hydrology and ecology.

## Background & Summary

Accurate modeling of hydrological, ecological, and climatic processes^1–3^, requires reliable spatial and temporal estimates of meteorology^2^. Near-surface observation-based meteorological datasets are ideal inputs for the aforementioned modeling applications^4,5^. However, meteorological observations are irregularly spaced^6^ and often clustered around population centers, while models typically require meteorological data on a quasi-continuous, regular grids, representing a practical challenge. Here, we present gridded daily precipitation (P), minimum temperature (Tmin) and maximum temperature (Tmax) data for the island of Java—the first of its kind, high-resolution, station-based gridded meteorology product in Indonesia.

Precipitation over Java exhibits large seasonal variability in which the dry season, May-Oct, has greater inter-annual variability than the wet season, Nov-Apr^7^. The southwestern part of the island receives more annual rainfall than the northeastern region due to influences from the ocean-atmospheric circulation in the Indian Ocean^8^. Mountains across the island (from west to east) create additional orographically-driven precipitation variability^8^ and their relief form a series of small watersheds that drain to the south and north.

Existing gridded meteorological data products for Java include satellite-only datasets, reanalysis products, coarse resolution gauge datasets and products that combine these sources. The details of these products are presented in Table 1.

Despite the availability of the products in Table 1, they each have unique shortcomings. Satellite products tend to have large sensor errors due to their dependence on rainfall regime (character of the seasonal distribution of rainfall–the more the rainfall regime tends toward deep convection, the more accurate the satellite estimates are), short time record and lack of local data^9^. On the other hand, reanalysis products tend to be coarser in resolution—0.5°×0.5° or coarser^10^. The International Precipitation Working Group (IPWG) found the satellite-based TRMM3B42 produced relatively accurate high-resolution precipitation estimates for operational use^9^–although, it too suffers from a relatively short record and coarse spatial resolution (0.25°×0.25°) spanning the period 1998–2013. Conversely, APHRODITE provides daily data (0.25°×0.25°) for a longer period 1950–2007 (ref. 3). However, over Java, APHRODITE was derived from only 20 stations^3^. The most recently updated rainfall products available in the region are the Multi-Source Weighted-Ensemble Precipitation (MSWEP)^11^ and the Climate Hazards group Infrared Precipitation with Stations (CHIRPS)^12^. While MSWEP mixes satellite and reanalysis products, CHIRPS combines gauge, satellite and reanalysis precipitation^11–13^. MSWEP is available on 0.25°×0.25° grid—for the period 1979–2015 (ref. 11), while CHIRPS rainfall estimates running from 1981 up to present on 0.25°×0.25° grid^12^.

This dataset was developed at a 0.125°×0.125° resolution, spanning 1985–2014. This temporal period was selected to balance available data with a temporal period long enough to enable hydroclimate research. The grid size was chosen to maximize station coverage while representing spatial variability of watershed-scale natural processes in the predominantly small watersheds of Java, and was gridded from a much larger number of precipitation gauges (765 stations) than other products, e.g., APHRODITE ~20 stations. A study by [8] recently applied these data to drive a land-surface model (LSM) over Java which found that higher spatial resolution meteorological data produced better model performance and hydrologic process representation than coarser meteorology. Below we describe the station data, the gridding methodology, and present an evaluation of the gridded product using two existing products (MSWEP and CHIRPS) as well as with a cross-validation procedure.

## Methods

### Station data

**Figure 1:**
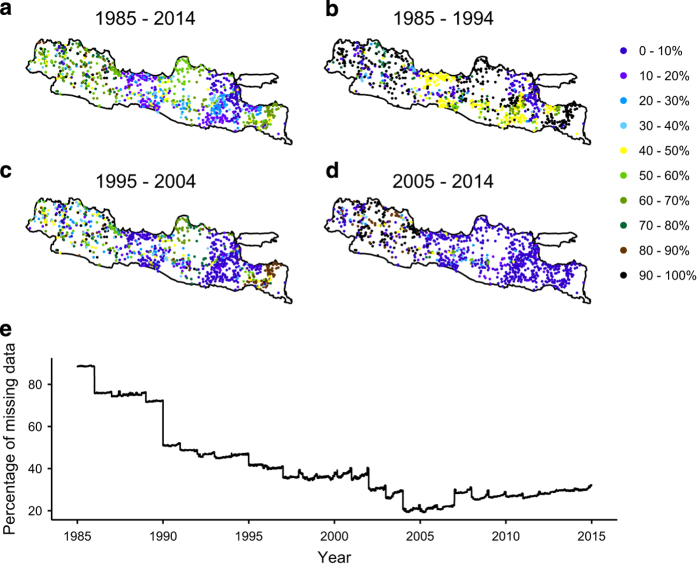
Spatial and temporal distribution of precipitation data. (**a**) the percentage of missing data for each station over the entire record period (the number of days with missing data divided by the total number of days), while (**b**), (**c**) and (**d**) show the percentage of missing data for the first, second, and third decade, respectively, and (**e**) the percentage of stations with missing daily data through time.

Figure 1 shows the spatial and temporal availability of precipitation observations over Java for each decade starting in 1985. The precipitation stations are irregularly distributed over the island. However, the mean station density is approximately one station per 135 km^2^. As a compromise between including both long-term and spatially diverse stations, we selected stations with more than 3,650 days of non-missing data over the study period (Fig. 1a). East Java has the fewest non-missing data followed by Central and West Java (Fig. 1b,c,d). The period around 2005 has the fewest missing data across the island whereas missing data were markedly more widespread before 1990.

### Gap-infilling and gridding procedure

Gap-infilling was conducted to create serially complete station records to minimize discontinuities in the gridded product during periods of missing data. Several methods are typically applied to interpolate and infill rainfall in time (i.e., gap-infilling) and space^14–17^. The Inverse Distance Weighting (IDW) and geostatistical Kriging methods with all their respective variants are the two most widely employed and evaluated methods in literature^16–18^. IDW is a straightforward method that requires relatively few input data. On the other hand, Kriging can account for the spatial correlations between neighboring observations, and can incorporate covariates into the gridding process^17–19^. All Kriging variants perform better than IDW at a monthly time scale^18^. However, at a daily time step, both IDW and Kriging produce comparably small errors^17^. In addition, for small watersheds, IDW was shown to perform better than Kriging^20^. In tropical regions, IDW interpolation performed slightly better than Kriging^19^. Kriging requires estimation of variogram to describe the degree of spatial dependence of a spatial random field or stochastic process, which is notoriously difficult to fit with short data records and it can be problematic fitting a variogram for daily fields like precipitation that can take on ‘zero’ values.

Given that the majority of watersheds in Java are small, IDW was chosen here for both gap-infilling and the gridding process. The interpolated values were estimated as a weighted linear combination of nearest observations, with the weights proportional to the inverse of the distance between neighboring observations and interpolation location^21^, as presented below.

(1)qˆ=∑i=1n1riαqi∑i=1n1riα

where $\hat{q}$ is the interpolated (gridded) value, *q*_*i*_ is the observed value in station *i*, *r*_*i*_ is the euclidean distance between interpolated station and station *i*; *α* is the power of distance and *n* is the total number of stations interpolated per grid.

**Figure 2:**
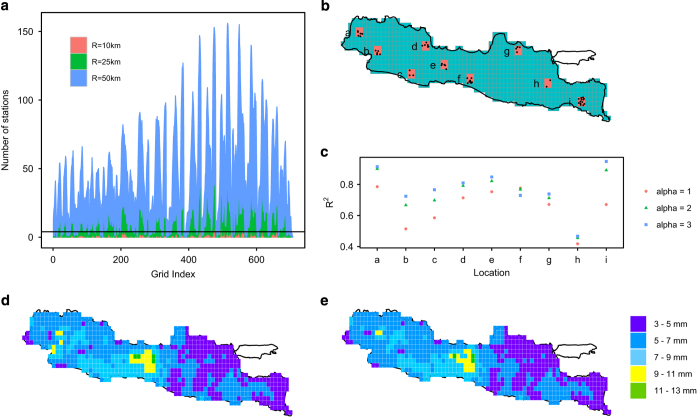
Selection of interpolation method. (**a**) the number of stations with non-missing data within radius of influence (*r*) of 10, 25 and 50 km for each grid where each grid was assigned an index/number, the horizontal black line is a threshold of 4 stations for IDW interpolation; (**b**) selected locations to examine the performance of interpolation, the grid size is equivalent to 0.25°×0.25° to capture at least one observed station; (**c**) model performance between the box-average gridded and observed precipitation computed as the square of the Pearson correlation coefficient, R^2^; (**d**) and (**e**) are mean daily precipitation which gridded using *r* of 25 and 50 km respectively.

The radius of influence (*r*) and the power of distance (α) are parameters which were adjusted to obtain an optimal interpolated value. Interpolated root mean square errors are minimized when *r* ranges from 10–30 km and α between 0–5 (ref. 22). To optimize, we considered *r* values of 10, 25 and 50 km to interpolate surrounding stations and α of 1, 2 and 3 to weigh local and regional influences. Since missing data were numerous at times (especially before 1990) and the distances between stations in some regions are relatively large (Fig. 1e), we added a constraint requiring a minimum number of stations to be interpolated. Four stations with non-missing data were required to lie within the radius (*n*=4), otherwise the nearest next-closest stations with non-missing data were selected. Figure 2a shows that the number of stations with non-missing data within *r* of 10 km was less than four, which fails to meet the threshold, thus only *r* of 25 and 50 km were applied, with a smaller radius (25 km) preferable to avoid smoothing of data from differing precipitation events and orographic facets.

To identify an α value which optimally captures spatial and temporal precipitation variability, we applied IDW to daily station precipitation as well as mean daily precipitation at 9 selected test locations spread relatively uniformly about the domain (Fig. 2b) using *r* of 25 km and α of 1, 2 and 3. The variability of station elevation was incorporated by defining R as the three-dimensional Euclidean distance—i.e., square root of sum of squared x, y and z. For each location, the spatial average of interpolated and observed daily precipitation were calculated and the correlations were computed. Figure 2c shows that in nearly all cases, α of 3 achieves the best performance. Using α of 3, we assessed the sensitivity of *r* to the interpolated values. IDW was less sensitive to radius of influence, as indicated by the similar spatial pattern of mean daily gridded precipitation between *r* of 25 versus 50 km (Fig. 2d,e). This is a feature of IDW whereby the higher the value of α, the less weight is apportioned to more distant stations. Accordingly, we ultimately elected to use α=3 and *r*=25 km—i.e., using more heavily weighting fewer stations, but retaining the ability to capture information from more distant.

### Code availability

The data were processed using a standard version of the R software, R.3.2.2. The code is publically available alongside the dataset (Data Citation 1). Some R packages need to be installed before implementing the code and full instructions are provided together with the code.

## Data Records

The various data sources are presented in Table 2. The Center of Water Resources Development and Management, Ministry of Public Works, Indonesia provided most of the daily precipitation data. Additional daily precipitation, minimum temperature and maximum temperature were obtained from the Bureau of Meteorology, Climatology and Geophysics of Indonesia. The availability of precipitation data was ultimately used to determine the most appropriate temporal domain for the dataset.

The final dataset contains gridded station data for precipitation, maximum and minimum temperature at a daily time step running from 1 January 1985–31 December 2014 at 0.125°×0.125° resolution. The dataset is stored in network common data form (NetCDF), archived at Data Citation 1.

## Technical Validation

We validated the gridding procedure at station, watershed and island scales over daily and monthly time steps using station observations as validation. We experimented with blending the station rainfall with the most updated existing rainfall products, MSWEP and CHIRPS, to explore the potential improvements for using a smooth surface (from MSWEP and CHIRPS) as the background for interpolation rather than exclusively gridding irregularly spaced stations. Further, the two products were developed using different sources and interpolation techniques^11,12^, potentially offering useful independent information. These are rainfall products notably overlap the entire period of data presented here. We first cross-validated the IDW procedure by removing daily observed values at each station and then interpolating neighboring stations—using the IDW method, i.e., cross-validation. The interpolated value could then be evaluated against the withheld observation from that day, we refer this as ‘station-IDW’. We repeated the cross-validation at randomly selected stations (15% of total stations) and included 2 additional rainfall combinations to explore the potential utility of including MSWEP and CHIRPS, e.g.,(i) station-IDW and MSWEP; and (ii) station-IDW and CHIRPS. These combinations were computed by averaging station-IDW with MSWEP and CHIRPS, respectively. Percent bias (PBIAS)—the difference between observation and interpolated estimates divided by the observation and reported as a percentage—was computed for daily precipitation to validate the goodness of fit of the interpolation procedure and data sources for the 15% of selected stations over the entire time period. Negative PBIAS indicates that the gap-filling and gridding overestimates the observation and vice versa, with an optimum value of zero. We ran 10 samples of random stations. For each sample, we calculated the spatial mean and s.d. of PBIAS for all combinations (Table 3).

As shown in Table 3, the mean and standard deviation of PBIAS for station-IDW are smaller (closer to zero) than other combinations with the exception of the standard deviation of Sample 4 where the smallest values are produced by averaged station-IDW and MSWEP. This indicates that the IDW method, applied to ground-based observation rainfall produces the best rainfall estimate for Java. We suspect that suspect that the blended products (MSWEP and CHIRPS) are best applied to larger scales and that the reason that station-IDW performs better, is because it is derived from many more stations.

**Figure 3:**
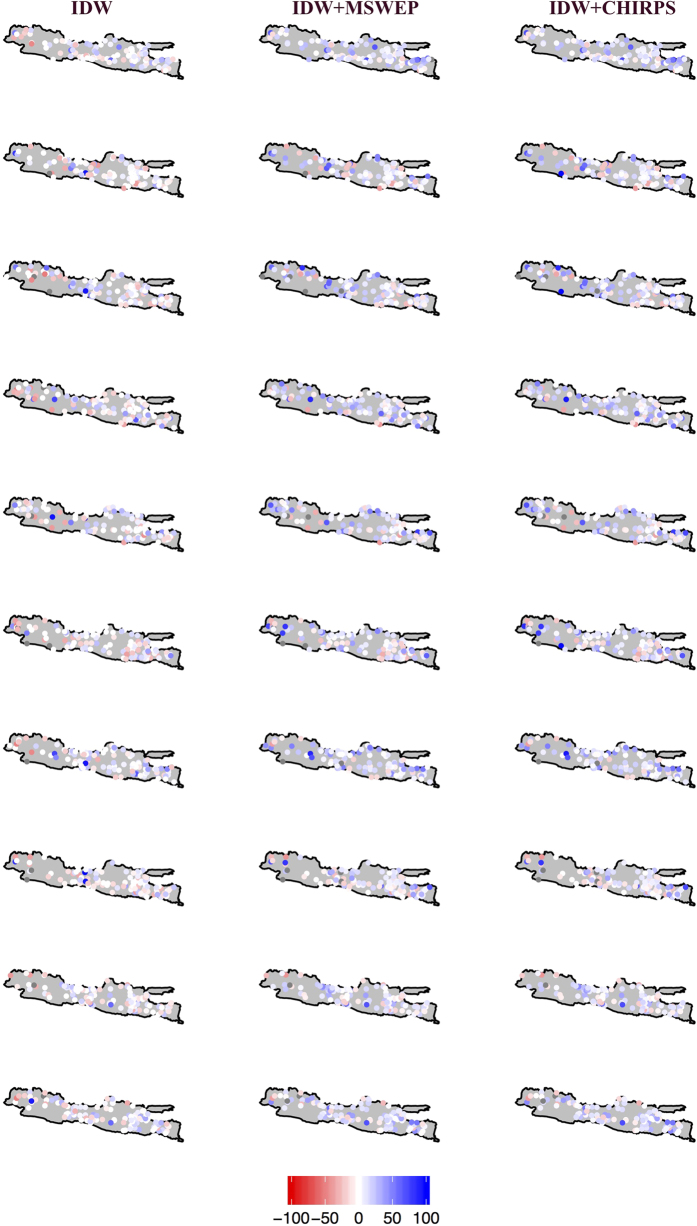
Spatial map of cross-validation PBIAS for three rainfall interpolation procedures. Station-IDW (left column), station-IDW+MSWEP (middle column) and station-IDW+CHIRPS (right column). Each row represents different random sampling of rainfall stations (15% of total stations).

Figure 3 shows a spatial map of PBIAS for cross-validation of the three combinations. For all samples, the station-IDW rainfall estimate has smaller errors than combination using MSWEP and CHIRPS. However, all three combinations exhibit similar patterns of overestimation near mountainous areas, indicating persistent errors in quantifying orographic gradients.

**Figure 4:**
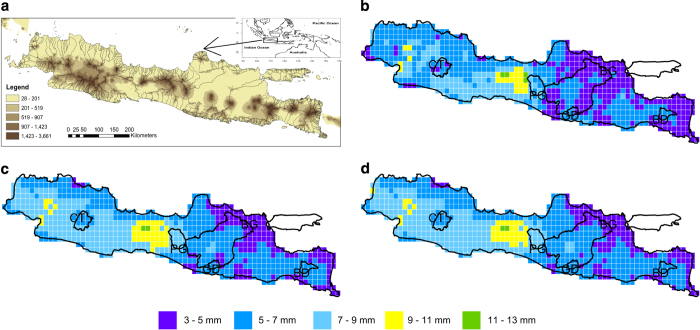
Watershed-scale validation. (**a**) topography of Java Island overlain by all watersheds; (**b**), (**c**) and (**d**) station-IDW, station-IDW+MSWEP, station-IDW+CHIRPS, respectively, overlain by 5 study watersheds.

At the watershed scale, station-IDW (Fig. 4b) exhibits more variability relative to other combinations (Fig. 4c,d). Distinctive differences in rainfall between watersheds such as PG, GD and BD can be seen for all three combinations. However, the MSWEP and CHIRPS blends have reduced variability relative to IDW-station. Given the similarities in both blended precipitation products and their reduced sensitivity to elevation, it is expected that station-IDW rainfall reconciles spatial variability more explicitly, as it contains information from many more stations than the other products and is gridded at a finer resolution.

**Figure 5:**
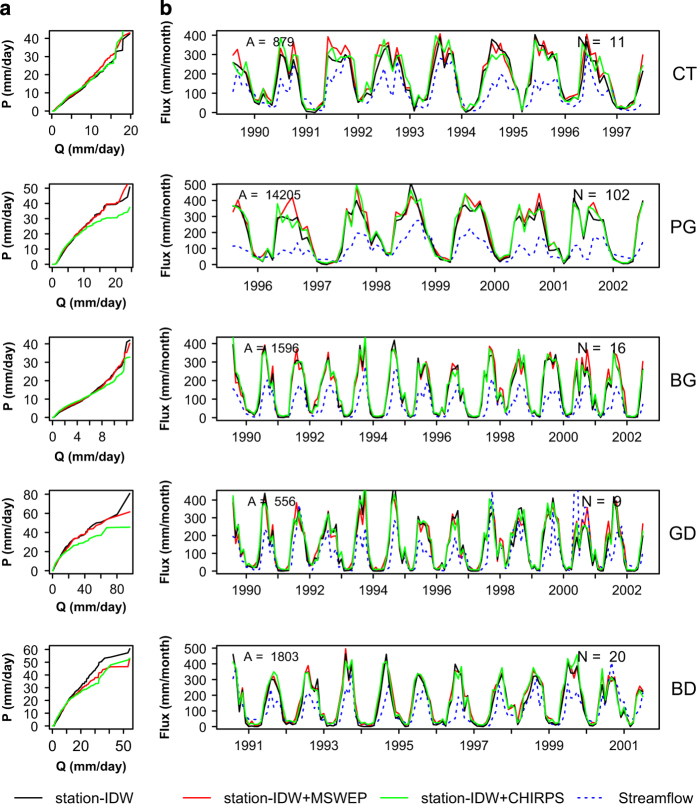
Watershed-scale interpolation performance. (**a**) Q-Q plots for daily gridded precipitation and observed streamflow of all combinations (station-IDW, station-IDW+MSWEP, station-IDW+CHIRPS) for five study watersheds, and (**b**) monthly hyetographs and hydrographs for the same watersheds and combinations, A is the watershed area, km^2^ and N is the number of grid cells in each watershed.

Figure 5 illustrates watershed-scale variability between gridded precipitation at daily and monthly time steps for each product combination. We validate the gridded precipitation with observed streamflow to underscore the importance of the water balance for understanding hydrologic processes, as discharge, Q, represents an integration of precipitation over an entire watershed^22,23^. We compare statistical features of daily gridded precipitation and observed streamflow in Citarum (CT), Progo (PG), Bengawan (BG), Grindulu (GD) and Bedadung (BD) watersheds. These watersheds have discharge that passed quality control procedures outlined by [8]. We use Q-Q plot to match the quantiles between the observed and interpolated esitmates and to identify the presence of outliers. As shown in the Q-Q plots, both gridded precipitation and observed streamflow have similar distributions with slight differences between station-IDW and averaged CHIRPS in PG and GD. The monthly gridded precipitation hyetograph follows the streamflow hydrograph in all the watersheds, with a few exceptions such as the earlier years of PG, while the year 2001 at GD likely has erroneous discharge, as it greatly exceeds precipitation. All three combinations have similar hyetograph shape. Overall, these features characterize the key hydrologic processes in Java, minimal lag time between monthly precipitation and streamflow^24^.

**Figure 6:**
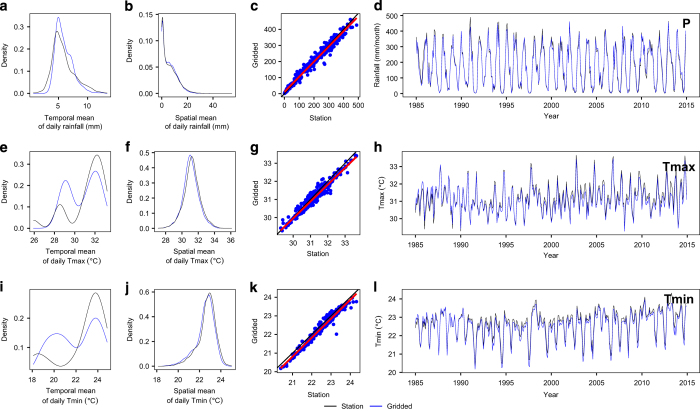
The probability density function (PDF) of mean daily P, Tmax and Tmin. (**a**,**e**,**i**), respectively, computed using the island-average value for each day in the record; the PDFs of mean daily P, Tmax, Tmin (**b**,**f**,**j**) computed across the mean daily value for each grid cell; the third column (**c**,**g**,**k**) shows scatterplots between daily gridded and observed P, Tmax and Tmin respectively overlaid by the 1:1 line (black) and regression line (red); and the fourth column (**d**,**h**,**l**) are monthly time series of P, Tmax and Tmin respectively.

An additional island-scale validation was conducted, comparing probability density functions (PDFs), scatterplots, and time series of station and gridded data for P, Tmin and Tmax. Both spatial (Fig. 6a,e,i) and temporal (Fig. 6b,f,j) PDFs were estimated, with the former computed using the mean island-wide field for each day, and the latter computed as the mean inter-grid-cell difference averaged over time. In addition, the daily and seasonal variability of the dataset was examined in scatterplots (Fig. 6c,g,k) and comparing their monthly time series (Fig. 6d,h,l). Given the relative similarity of the three combinations shown already at station and watershed scales, we perform the remaining island scale analyses using station-IDW only.

The gridded and station-based precipitation are comparably distributed over time and space with similar spatial PDF features for minimum and maximum temperature. However, the temporal PDFs for temperature exhibit fairly large differences, which we attribute to large distances between temperature stations. Overall, scatterplots and monthly time series of the spatial average of meteorological fields indicate that the gridded product closely matches the station data for all variables, with regression lines close to the 1:1 line (black line) (Fig. 3c,g,k).

## Usage Notes

We caution data users who aim to use this dataset for trend analyses that many stations do not extend for the entire period 1985–2014. Another point of caution is that the pre-1990 period has greater than 50% of stations reporting missing data. Similarly, users of data in West Java particularly in the period of 1985–1994 and 2005–2014 should be aware that most stations have more than 70% missing data.

## Additional Information

**How to cite this article:** Yanto, *et al.* Development of a gridded meteorological dataset over Java island, Indonesia 1985–2014. *Sci. Data* 4:170072 doi: 10.1038/sdata.2017.72 (2017).

**Publisher’s note:** Springer Nature remains neutral with regard to jurisdictional claims in published maps and institutional affiliations.

## Supplementary Material



## Figures and Tables

**Table 1:** Meteorological data products available for Java Island.

**Source**	**Product**	**Spatial resolution**	**Reference**
Gauge datasets	Climatic Research Unit Time Series (CRUTS)	0.5°×0.5°	^25^
	Global Precipitation Climatology Centre (GPCC)	0.5°×0.5°	^26^
	Asian Precipitation—Highly—Resolved Observational Data Integration Towards Evaluation of Water Resources (APHRODITE)	0.25°×0.25°	^3^
Satellite-only datasets	CICS High-Resolution Optimally Interpolated Microwave Precipitation from Satellites (CHOMPS)	0.25°×0.25°	^27^
	Precipitation Estimation from Remote Sensing Information using Artificial Neural Network (PERSIANN)	0.25°×0.25°	^28^
	Tropical Rainfall Measuring Mission Product 3B42 (TRMM3B42)	0.25°×0.25°	^29^
Reanalysis products	Global Precipitation Climatology Project (GPCP)	1°×1°	^30^
	CPC Merged Analysis of Precipitation (CMAP)	2.5°×2.5°	^31^
	Modern Era Retrospective Analysis for Research and Applications (MERRA)	0.5°×0.5°	^32^
Blended source datasets	Multi-Source Weighted-Ensemble Precipitation (MSWEP)	0.25°×0.25°	^11^
	Climate Hazardss group Infrared Precipitation with Stations (CHIRPS)	0.25°×0.25°	^12^

**Table 2:** Summary of daily data sources used to develop the meteorological dataset.

**Data type**	**Source**	**Details**
Precipitation	Center for Water Resources Research and Development, Ministry of Public Works, Indonesia	945 total stations with 746 stations satisfying the minimum length constraint of 3,650 days
	BMKG Indonesia	19 total stations and 16 stations satisfy minimum length of 3,650 days
Minimum temperature	BMKG Indonesia	19 total stations and 16 stations satisfy minimum length of 3,650 days
Maximum temperature	BMKG Indonesia	19 total stations and 16 stations satisfy minimum length of 3,650 days

**Table 3:** Error statistics of randomly 106 stations sampled for cross-validation.

**Sample**	**Mean of PBIAS**			**Standard deviation of PBIAS**		
	**Station-IDW**	**Station-IDW and MSWEP**	**Station-IDW and CHIRPS**	**Station-IDW**	**Station-IDW and MSWEP**	**Station-IDW and CHIRPS**
1	**3.04**	13.26	13.41	**16.60**	21.08	21.34
2	**2.06**	9.95	9.90	**21.92**	25.79	25.12
3	**8.78**	17.87	17.92	**40.16**	43.60	43.56
4	**2.25**	11.02	11.25	26.71	**25.57**	25.79
5	**3.24**	11.98	11.85	**23.70**	29.22	29.24
6	**3.38**	12.83	13.01	**26.10**	31.39	31.96
7	**10.55**	20.88	20.65	**56.30**	69.46	67.63
8	**2.96**	10.25	10.58	**25.28**	29.92	29.85
9	**3.55**	10.71	10.60	**33.72**	36.56	36.39
10	**4.35**	10.71	11.62	**24.43**	25.08	25.38
